# The Effects of Home-Based Cognitive Training on Verbal Working Memory and Language Comprehension in Older Adulthood

**DOI:** 10.3389/fnagi.2017.00256

**Published:** 2017-08-08

**Authors:** Brennan R. Payne, Elizabeth A. L. Stine-Morrow

**Affiliations:** ^1^The Beckman Institute for Advanced Science and Technology, University of Illinois at Urbana-Champaign, Urbana IL, United States; ^2^Department of Psychology, University of Utah, Salt Lake City UT, United States; ^3^Department of Educational Psychology, University of Illinois at Urbana-Champaign, Urbana IL, United States

**Keywords:** cognitive training, working memory, language, aging

## Abstract

Effective language understanding is crucial to maintaining cognitive abilities and learning new information through adulthood. However, age-related declines in working memory (WM) have a robust negative influence on multiple aspects of language comprehension and use, potentially limiting communicative competence. In the current study (*N* = 41), we examined the effects of a novel home-based computerized cognitive training program targeting verbal WM on changes in verbal WM and language comprehension in healthy older adults relative to an active component-control group. Participants in the WM training group showed non-linear improvements in performance on trained verbal WM tasks. Relative to the active control group, WM training participants also showed improvements on untrained verbal WM tasks and selective improvements across untrained dimensions of language, including sentence memory, verbal fluency, and comprehension of syntactically ambiguous sentences. Though the current study is preliminary in nature, it does provide initial promising evidence that WM training may influence components of language comprehension in adulthood and suggests that home-based training of WM may be a viable option for probing the scope and limits of cognitive plasticity in older adults.

## Introduction

Literacy and effective language comprehension are crucial to maintaining cognitive abilities and learning from text through adulthood (Manly et al., 2003; Stern, 2009; Payne et al., 2012a; Stine-Morrow et al., 2015). However, normative age-related cognitive changes have a profound influence on language understanding, especially for effortful comprehension and memory processes (Wingfield and Stine-Morrow, 2000; Wlotko et al., 2010; Payne and Stine-Morrow, 2016; Stine-Morrow and Payne, 2016). Working memory (WM) —the ability to temporarily store, maintain, and organize task-relevant information— is often implicated as a domain-general mechanism responsible for such age-related changes in language understanding (Stine and Wingfield, 1987; Van der Linden et al., 1999; Borella et al., 2011; Kemper, 2012; Payne et al., 2014a). Although virtually all models of language comprehension include some mechanism to account for WM constraints (see Pickering and van Gompel, 2006; Caplan and Waters, 2013 for reviews), the degree to which the WM system directly supports comprehension and the role of WM in language understanding is a topic of ongoing debate (see, e.g., Just and Carpenter, 1992; Carpenter et al., 1994; Caplan and Waters, 1999, 2013; Just and Varma, 2002, 2007; MacDonald and Christiansen, 2002).

The majority of research examining the influence of WM on language comprehension has relied on either dual-task paradigms to examine the effects of manipulated WM constraints on language comprehension (Smiler et al., 2003; Fedorenko et al., 2006; Kemper and Herman, 2006), or correlational approaches that test the relationship between individual differences in WM and language comprehension (King and Just, 1991; Just and Carpenter, 1992; Caplan and Waters, 1999; DeDe et al., 2004; Stine-Morrow et al., 2008; Noh and Stine-Morrow, 2009; Caplan et al., 2011; Payne et al., 2014a). In contrast, the current study used home-based cognitive training as an experimental approach to examining the degree to which the verbal WM system underlies language comprehension (cf. Novick et al., 2013; Hussey et al., 2016).

### Aging, Working Memory, and Language Comprehension

Working memory limitations have historically been invoked in models of language understanding to explain comprehension difficulties for linguistically complex material (Miller and Chomsky, 1963). Current research activity has focused on performance on complex WM span tasks, which have been argued to underpin performance on a wide range of both complex and everyday tasks (Engle, 2010; Baddeley, 2012). While there are many contemporary models of WM, each of which make slightly different predictions or have slightly different foci (e.g., Cowan, 2000; Engle, 2002, 2010; Kane et al., 2007a,b), most models converge on a similar account that the WM system supports “the ability to simultaneously maintain information in an active and readily accessible state, while concurrently and selectively processing new information…” (Conway et al., 2007; p. 3). Complex WM span measures such as the *reading span* (Daneman and Carpenter, 1980; Wingfield et al., 1988) and the *operation span* (Turner and Engle, 1989) task share the requirement to simultaneously hold information in memory while performing some concurrent processing. This dual-task nature of complex span tasks critically sets them apart from simple STM tasks that are not predictive of higher-order cognition (reviewed in Baddeley, 2012). In contrast, performance on complex WM span tasks predict individual differences in a number of higher-order cognitive abilities including reasoning, episodic memory, attentional control, and intelligence (see Conway et al., 2007 for reviews). WM is also related to comprehension, with meta-analytic correlations ranging between *r* = 0.41 and *r* = 0.52 (Daneman and Merikle, 1996). Theoretical accounts of such relationships center on the reliance on WM for constructing, storing, retrieving, and integrating an incremental representation of the text’s meaning as decoding and parsing of the surface input is ongoing (e.g., Just and Carpenter, 1992; Gibson, 1998; Lewis and Vasishth, 2005).

Performance on complex span tasks declines with aging (e.g., Bopp and Verhaeghen, 2005), as does comprehension and memory for language (Kemper, 1987; DeDe et al., 2004; Payne et al., 2014b). For example, a meta-analysis by Johnson (2003) revealed that on average, older adults perform at about the 22nd percentile of the distribution of younger adults in text memory. Similar effect sizes for age-related declines in immediate language memory have been found in a longitudinal study tracking changes in older adults’ auditory discourse memory over a 10-year period (Payne et al., 2014b). Although there is considerable debate regarding the impact of WM deficits on on-line measures of real-time language processing in aging (Caplan and Waters, 1999; Kemper and Liu, 2007; Caplan et al., 2011; Payne et al., 2014a), verbal WM has been found to reliably mediate age-related changes in “off-line” measures of language comprehension and language memory (Kwong See and Ryan, 1995; Van der Linden et al., 1999; Hertzog et al., 2003; DeDe et al., 2004; Stine-Morrow et al., 2008; Borella et al., 2011).

Moreover, age differences in sentence comprehension accuracy are larger for sentences that are more semantically or syntactically complex, and these differences in performance have been found to be dependent upon individual differences in verbal WM capacity (Kemper, 1987; Stine and Hindman, 1994; Stine-Morrow et al., 2000; Christianson et al., 2006; Payne et al., 2014a). For example, “garden path” sentences such as (1) introduce a temporary syntactic ambiguity.

(1) *The experienced soldiers warned about the dangers conducted the midnight raid*.

Typically, the first verb *warned* is initially (and incorrectly) interpreted as the main verb of the sentence (rather than as the verb of the reduced relative clause), creating difficulty when the reader encounters the second verb *conducted*; resolution thus requires a revision of the initial analysis (Bever, 1970; Clifton et al., 2003), which entails maintaining the alternate parse of the sentence during processing. WM capacity is an important predictor of resolution in garden-path ambiguities in younger (Just and Carpenter, 1992; MacDonald et al., 1992; Just and Varma, 2002) and older (Kemtes and Kemper, 1997; Kemper et al., 2004; Christianson et al., 2006) adults, as well as in other syntactically complex constructions, such as object-relative clauses (Stine-Morrow et al., 2000; DeDe et al., 2004), and long distance dependencies (King and Kutas, 1995; Caplan et al., 2011; Payne et al., 2014a).

### Cognitive Training in Aging

Cognitive training has a long history in aging research, dating back over 30 years. Studies have reliably demonstrated that older adults show targeted improvements in trained abilities, including episodic memory, inductive reasoning, task switching, psychomotor speed, and WM capacity (Willis et al., 1981; Baltes and Willis, 1982; Willis and Nesselroade, 1990; Ball et al., 2002; Rebok, 2008; cf. Stine-Morrow and Basak, 2011; Rebok et al., 2014, for a review). Importantly, the demonstration that targeted training in a cognitive domain can improve performance in that domain in older adults is not trivial considering evidence of age-related declines in plasticity (Lövdén et al., 2010). At the same time, there is considerable debate regarding whether and how cognitive training may produce “far” transfer, that is, improvements on untrained tasks that are distal from the trained ability—with studies demonstrating variable effect sizes for transfer (Melby-Lervåg and Hulme, 2013, 2016; Melby-Lervåg et al., 2016; Simons et al., 2016; but see Karbach and Verhaeghen, 2014; Au et al., 2015, 2016; Greenwood and Parasuraman, 2016). Note that there is considerable variability in cognitive training programs as well as what constitutes a target of transfer across the literature (Kelly et al., 2014; Simons et al., 2016)—the focus of the current study is on whether targeted cognitive training in one domain can produce transfer *across other cognitive domains*. Training effects on other outcomes (e.g., instrumental activities of daily living, self-rated health; e.g., Rebok et al., 2014, see Kelly et al., 2014; Simons et al., 2016 for recent reviews) are beyond the scope of this study and are not discussed in further detail. In the cognitive aging literature, cognitive training has most reliably produced narrow transfer across untrained cognitive domains (see reviews in Stine-Morrow and Basak, 2011; Simons et al., 2016). For example, the ACTIVE trial (Ball et al., 2002; Willis et al., 2006; Rebok et al., 2014), was the largest cognitive intervention study to date (*N* = 2,832) and arguably remains the benchmark cognitive training study, conforming to many of the best practices for intervention research. Healthy older adult participants’ completed 10 sessions of training in either processing speed, episodic memory (targeting strategy use), or inductive reasoning. Although evidence of transfer to measures of functional and clinical outcomes (e.g., instrumental activities of daily living, depressive symptoms, driving mobility, and others) has been reported from ACTIVE (e.g., Willis et al., 2006; Wolinsky et al., 2010; Rebok et al., 2014), the effects of the transfer of cognitive training across cognitive outcomes was narrow, with large and maintained effects of training on measures proximal to the training (e.g., memory training improved episodic memory) with little evidence of transfer across other cognitive domains (e.g., memory training had no impact on processing speed or inductive reasoning).

Some researchers have noted that training regimens that target executive control and WM functions have shown more promise in stimulating cognitive improvements beyond near transfer and practice effects in older adults (Karbach and Verhaeghen, 2014; Greenwood and Parasuraman, 2016). For example, a number of studies have demonstrated that WM training increases performance not only on span tasks that are untrained but proximal to WM, but also some (limited) evidence for transfer to other cognitive domains such as inhibitory control, memory, and reasoning (Buschkuehl et al., 2008; Li et al., 2008; Borella et al., 2010, 2017; Brehmer et al., 2011; Richmond et al., 2011; Zinke et al., 2014, see Karbach and Verhaeghen, 2014 for a recent meta-analysis). On the other hand, there is an active debate regarding whether such WM training can produce reliable broad-based transfer across cognitive domains, such as transfer to fluid intelligence, in younger and older adults, with studies producing overall inconsistent results (e.g., Shipstead et al., 2012; Harrison et al., 2013; Melby-Lervåg and Hulme, 2013; Simons et al., 2016; but see Karbach and Verhaeghen, 2014; Au et al., 2015, 2016).

One limitation of these reviews and meta-analyses is that there is considerable heterogeneity in the tasks used to train and measure WM, making it difficult to evaluate efficacy in the aggregate (cf. Morrison and Chein, 2011; Shipstead et al., 2012; Melby-Lervåg and Hulme, 2013). At the same time, a full understanding of the effects of WM training has been obscured by a literature that is rife with methodological short-comings. Calls for improved methodological and quantitative standards in cognitive training research are abundant (e.g., Shipstead et al., 2012; Melby-Lervåg and Hulme, 2013; Walton et al., 2014; Simons et al., 2016). Some of the issues clouding the extant literature include the lack of adequate control groups and very small sample sizes. Moreover, any effect of improved WM on other abilities hinges on the assumption that these constructs rely on overlapping cognitive and neural resources that are engaged across multiple domains (cf. Dahlin et al., 2008; Hussey et al., 2016; Lindenberger et al., 2017) and yet there exists a surprising lack of consideration of theoretical mechanisms of training effects and transfer in the literature (cf. Shipstead et al., 2012). One recent attempt to elucidate the benefits of WM training in aging in the context of substantial heterogeneity of training outcomes came from Borella et al. (2017), who performed an integrative data analysis of four training intervention studies from their group that used an identical complex WM training protocol, the same target outcome measures, and similar samples of healthy older adults (total *N* across studies = 148). This study showed that, in aggregate, there was evidence for near transfer of WM training that was maintained for at least 6–8 months. In addition, they found evidence for immediate transfer of complex span training to measures of reasoning and processing speed, but also showed considerable individual differences in responsiveness to the training (cf. Payne et al., 2012b).

### The Current Study

The current study aimed to capitalize on the principles of vertical transfer to examine the degree to which training-related improvements in WM modulate targeted language comprehension functions that putatively rely heavily on WM. Older adults were randomly assigned to either a cognitive training program targeting complex verbal WM or an active control targeting decision speed, both of which were home-based programs delivered via electronic tablets.

Our goal was to address three key issues. First, we were interested in the extent to which WM, as a critical underpinning for language, is plastic and responsive to training-related improvements. Second, we wanted to test the causal hypothesis that WM capacity is a critical resource for language comprehension and memory. Manipulating WM capacity through training and examining its effects on language outcomes afforded the opportunity to directly examine the causal link that is often assumed based on correlational results (Daneman and Merikle, 1996). A number of studies have examined language comprehension as an outcome of cognitive training interventions (Chein and Morrison, 2010; Shiran and Breznitz, 2011; Carretti et al., 2013a,b; Novick et al., 2013; Karbach and Verhaeghen, 2014; Hussey et al., 2016) but nearly all of this work has focused on healthy young college adults or child populations with specific reading difficulties (e.g., Shiran and Breznitz, 2011; Karbach et al., 2015). Karbach et al. (2015) found evidence in children that adaptive WM training benefited standardized measures of reading comprehension but not measures of math performance or executive control (e.g., inhibition, task switching), suggesting a potentially unique pathway of WM training to comprehension. To our knowledge, only one study, by Carretti et al. (2013b), has specifically examined the effects of WM training on language outcomes among older adults. The training in this study consisted of multiple components, which included not only complex span tasks but also retrieval tasks incorporated into text processing tasks. As such, the improvements observed in language performance may have derived from direct instruction in components of reading comprehension rather than WM processes. In other words, the substantial overlap between the training and transfer task in the Carretti colleagues experiment makes it difficult to evaluate the isolated effects of WM improvement on language. Thus, there has not as yet been a definitive test of the hypothesis that WM training can modulate language performance in older adults, to our knowledge.

Finally, our goal was to develop a model of home-based training using technology that would both offer potential for scaling up for wider use and provide a medium for effective placebo control. In fact, home-based training in other domains has demonstrated good adherence and gains comparable to those observed in the laboratory in older adults (Margrett and Willis, 2006; Payne et al., 2012b; Stine-Morrow et al., 2014). Training tasks were designed to closely match the properties of complex verbal WM tasks in a mobile electronic format that was not only appealing for users, but also provided a detailed record of adherence to the study protocol as well as daily performance gains. We contrasted this with an active-component control group that was comparable to the training task in surface features, feedback, and engagement. Outcomes were measures of complex span tasks that were not directly trained as well as tasks that assessed various aspects of language performance. We were specifically interested in the degree to which WM training would impact immediate memory for sentences, comprehension of sentences that differed in their syntactic complexity, and discourse comprehension and memory.

## Materials and Methods

### Participants

Volunteers were recruited from the Champaign-Urbana community through flyer advertisements, information booths at the farmer’s market and related events, e-mail lists, and through phone recruitment from a database of older adult volunteers in the community who had previously participated in studies at the Beckman Institute.

**FIGURE 1:**
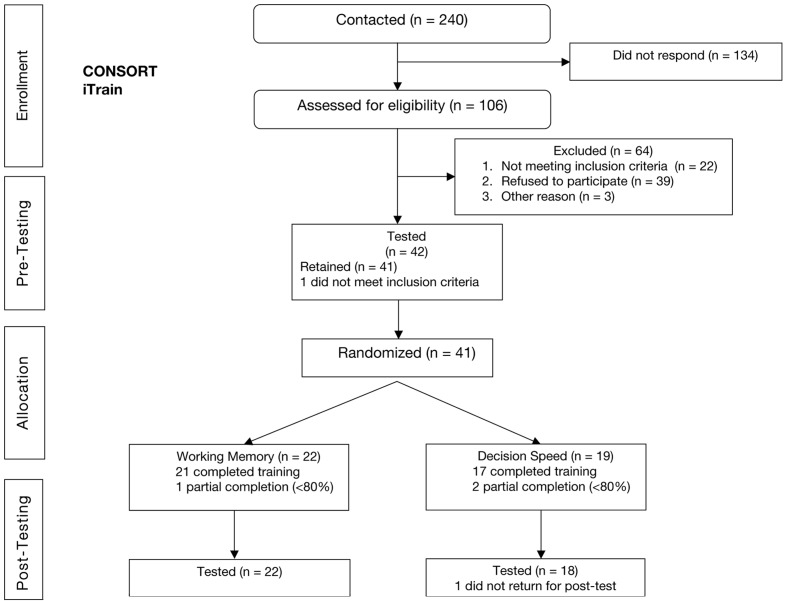
CONSORT diagram for iTrain study.

A CONSORT (CONsolidated Standards of Reporting Trials) diagram is presented in **Figure 1** (Altman et al., 2001), which provides a graphical representation of the recruitment process and the flow of participants through the study, from eligibility to post-testing. A total of 240 individuals were contacted either by phone or e-mail from our recruitment database, or after expressing interest in the study. Of those, 134 did not follow-up or reply to our invitation to participate in the study. A total of 106 individuals were then assessed for eligibility. Participants were required to be 60 years of age or older, native English speakers with no exposure to other languages before the age of five, normal or corrected-to-normal vision (self-reported), no history of cancer treatment, closed head injury, or traumatic brain injury, no history of Alzheimer’s disease, Parkinson’s disease, Schizophrenia, or other neurological or psychiatric disorders, not currently taking any psychoactive medications (e.g., anti-depressant, anti-anxiety, anti-seizure), and had not participated in a physical, social, or cognitive intervention study within the previous 3 years. Of those assessed for eligibility, 39 refused to continue participation after learning more about the study, 22 were excluded for not meeting one or more of the inclusion criteria above (*N* = 9 recently participated in an intervention study; *N* = 8 self-reported history of neurological or psychiatric disease; *N* = 4 currently taking a psychoactive medication; *N* = 1 did not meet the age requirement), and three were excluded for other various reasons (e.g., loss of contact, restrictive scheduling constraints).

Thus, a total of 42 individuals were pre-tested. One participant did not meet inclusion criteria at baseline, based on an inability to complete the pre-test cognitive assessment. Thus 41 individuals were randomly assigned to either a treatment (*n* = 22) or control (*n* = 19) group. Of those, 21 in the training group, and 17 in the control group, completed at least 80% of the training sessions. **Table 1** presents demographics at baseline in the control and treatment groups. As can be seen in **Table 1**, differences between the two groups in age, *t*(39) = 0.29, education, *t*(39) = 0.53, sex, χ^2^(1) = 0.005, MoCA score (a clinical tool used for assessing risk for mild cognitive impairment, Nasreddine et al., 2005), *t*(39) = 0.42, and vocabulary score (ETS extended range vocabulary task administered at baseline only), *t*(39) = 0.78 were negligible. Importantly, we adopted an intention-to-treat analysis approach (Hollis and Campbell, 1999; Gupta, 2011), whereby individuals who did not complete the training were actively recruited to participate in post-testing and were included in all analyses. This method results in a conservative test of the treatment effect by de-confounding any potential treatment effects on outcome measures truly due to non-adherence.

**Table 1:** Baseline demographics in control and treatment groups.

	Control		Treatment			
				
	*M* or count	*SD* or %	*M* or count	*SD* or %	*Diff*	*95% CI*
(1) Age	68.11	6.24	67.68	2.77	-0.42	[-2.55, 3.41]
(2) Years of education	17.47	2.38	17.09	2.20	-0.38	[-1.83, 1.07]
(3) MoCA	27.21	2.39	27.77	1.93	0.56	[-0.81, 1.92]
(4) Vocabulary	0.63	0.23	0.68	0.18	0.04	[-0.09, 0.17]
(5) % Female	14	74%	16	73%	0.01	[-0.26, 0.26]


*MoCA is Montreal Cognitive Assessment (Maximum = 30, Range = 24–30). Vocabulary is scored as the proportion of accurate items on the ETS Extended Range Vocabulary Test. Diff = mean difference between groups at baseline. There were no significant differences between the treatment and control groups at baseline.*

### Experimental Design and Overview

A pretest–postest randomized controlled experimental design with an active control group was used to examine the effects of WM training. Participants were asked to complete a total of five 30-min sessions in each week, for a total of 15 sessions over a 3-week period (or 7.5 h of total training). The interval between pre-test and post-test sessions was held constant across participants such that post-testing occurred no more than 4 weeks from the pre-test dates.

### Experimental Groups

#### Working Memory Training

A novel home-based complex verbal WM training program called iTrain was designed for the study. It was written in Objective-C and implemented for use on iPad tablet computers via the Xcode environment. The program was designed for home-based training to allow participants to complete training sessions without having to make daily visits to the lab while also allowing us to monitor adherence. Prior studies suggest that home-based cognitive training shows gains on the same order of magnitude as lab-based training (Margrett and Willis, 2006; Stine-Morrow et al., 2014), and also results in high retention rates in healthy older adults in part because participants do not have to travel to the lab daily throughout the course of the intervention.

The three tasks in iTrain – Category Span, Lexical Decision Span, and Sentence Span— were designed to exercise verbal WM by requiring a dual-task load of concurrent language processing and memory storage. In the *Category Span task*, participants were presented with a semantic category at the top of the screen (e.g., weather) along with a set of single words for which they made validity judgments (e.g., humidity – Yes; chocolate – No). Each trial consisted of the category and target word presented for 4 s. At any point within this duration, participants could decide if the target matched or did not match the category by pressing a “Yes” button or “No” button at the bottom of the screen. Once participants made a decision, the target word would disappear and participants would be presented with accuracy feedback (a green check mark if correct, or a red cross if incorrect) for 1 s. The program would then progress to the next trial within the set. If participants took longer than 4 s to respond, then the target word would disappear to prevent using extra time to develop artificial encoding strategies. However, participants could still respond. If participants failed to respond after a total of 4 s from the target word offset, then the trial would be marked as incorrect and the next trial within the set would begin. At the end of each set, participants were cued to recall each of the words in the order in which they were presented. The cued recall screen consisted of a set of empty text boxes that participants could press and then type their responses via an on-screen keyboard. Participants had no time limit to enter their recall responses at the prompt. Categories and exemplars were drawn from the Van Overschelde et al. (2004) category norms. The final stimulus set included a total of 69 unique categories and over 1500 unique words. Items were drawn randomly such that, within a set, each word had an equal probability of belonging to the presented category or not. Across training sessions, items were rotated through such that all categories had to be selected at least once before a particular category could be repeated again.

In *Lexical Decision Span*, participants were presented with a set of letter strings constituting words (e.g., seek) or non-words (e.g., ceek) and were cued to decide whether or not each string formed a word or not. The letter string was presented for 4 s. At any point within this interval, participants could decide if the letter string was a word or non-word by pressing a “Yes” button or “No” button at the bottom of the screen. Once participants made a decision, the letter string would disappear, and participants were presented with accuracy feedback (a green check mark if correct, or a red cross if incorrect) for 1 s. If participants took longer than 4 s to respond, then the letter string would disappear, but participants could still respond. However, if participants failed to respond after a total of 4 s from offset of the letter string, then the trial would be marked as incorrect and the next trial within the set would begin. Following each lexical decision, an unrelated single letter was presented for 1500 ms for participants to recall at the end of the set. At the end of each set, participants were cued to recall each of the letters in the order in which they were presented. The cued recall screen consisted of a set of empty text boxes that participants could press and then type their responses via an on-screen keyboard. Participants had no time limit to enter their recall responses at the prompt. A total of 9,000 common and proper nouns and 10,000 phonologically regular and pronounceable non-words were generated from the English Lexicon Project database (Balota et al., 2007). Word/non-word strings ranged in length between 4 and 9 characters (for word stimuli: log word frequency range: 5–13.67).

Finally, in *Sentence Span*, participants read a series of either semantically congruent sentences or “syntactic prose” sentences (e.g., *As the ship gets better, your child needs to develop this oven*) for which they made sentence acceptability judgments on each sentence (cf. Waters and Caplan, 1996, 2003). Participants had 15 s to read each sentence and make an acceptability judgment by pressing a “Yes” button or “No” button at the bottom of the screen. Once participants made a decision, the sentence would disappear, and participants were then presented with accuracy feedback (a green check mark if correct, or a red cross if incorrect) for 1 s. If participants took longer than 15 s to respond, then the sentence would disappear. If participants failed to respond after a total of 5 s from the offset of the sentence, then the trial would be marked as incorrect and the next trial within the set would begin. At the end of the set, participants were cued to recall the last word of each sentence in the order in which they were presented. The cued recall screen consisted of a set of empty text boxes that participants could press and then type their responses via an on-screen keyboard. Participants had no time limit to enter their recall responses at the prompt. Acceptable sentences were adapted from two sources. The Nelson and Narens (1980) general information question norms provided 244 sentences. The other source was the Manually Annotated Sub-Corpus (MASC) of the Open American National Corpus (Ide et al., 2013), which provided 301 sentences that ranged widely in topic, length, and syntactic structure. In addition, 346 unacceptable sentences were adapted from the “syntactic prose” conditions in earlier studies by Lee and Federmeier (2011) and Payne et al. (2015). Unacceptable sentences have syntactically well-formed sentence frames, but contain no coherent message-level semantics. All sentences ranged between 60 and 90 characters, and all sentence final words were between 4 and 9 characters.

The training was designed to be individually adaptive (cf. Lustig et al., 2009; Karbach et al., 2015), such that for all three tasks, the set size (number of items to recall within a trial) adaptively changed according to current performance. In this way, each participant was always engaging in the task at a level that was matched to his or her current ability. Task difficulty was programmed to follow a step function, such that when recall was perfect on set size *n*, the set size for the next set was increased to *n*+1. If perfect recall was not achieved at set size *n*, the set size was reduced to *n*-1. At the end of each set, feedback was presented to participants on both the accuracy of the judgment task (proportion correctly judged) and the proportion of items correctly recalled. The presentation order of the three tasks was randomized across session. The memory set size on the first session began at *n* = 2 for all tasks and subjects. The memory set size at the end of each training session was saved so that participants began the following session at the memory set level from their prior training session. All timing and set size parameters were based on extensive norming and testing of the iTrain software during its development. The source code for the training can be viewed and downloaded in full at: https://github.com/TALL1532/itrain.

#### Active Component-Control Group

A component-control design (Mohr et al., 2009; Boot et al., 2013) was adopted in designing the active control group. In a component-control design, a multi-component intervention serves as the focal treatment and an active control group is created by administering the same treatment absent a single component of the focal training. By matching the two groups on the surface level aspects of the tasks, along with presenting the same stimuli, this process reduces the likelihood of placebo effects or differential expectancies for change (Boot et al., 2013).

Participants in the active control group completed the same three tasks as in the treatment group without the recall component. Thus, in the *Category Task*, participants practiced making speeded category judgments; in the *Lexical Decision Task*, participants practiced making speeded lexical decision judgments; and in the *Sentence Task*, participants practiced making speeded semantic acceptability judgments. The items were identical to the WM training. Importantly, both the treatment and control groups were matched in their exposure to stimuli, the absolute magnitude of time allocated to training (15 30-min sessions over 3 weeks), and the amount and type of linguistic exposure. Thus, findings comparing the treatment and active control groups are controlled for exposure to linguistic stimuli, an important factor given the putative relationship between verbal WM and language experience (cf. MacDonald and Christiansen, 2002; Wells et al., 2009; Payne et al., 2012a, 2014a). Because removing the memory load from the WM training makes the task less demanding and potentially less engaging, an individually adaptive speed threshold was added in order to maintain continued interest in the task, de-confound memory load from task adaptivity, and reduce the potential for differences in expectancy for training benefits in the two groups (Boot et al., 2013).

In the control training, participants were presented with stimuli in blocks of 15 items and told to make their judgments (lexical decision, category, sentence acceptability) as quickly as possible. The starting presentations times for each task were identical to the presentation times in the WM training task (described above). However, as participants improved in accuracy in the judgment decisions, presentation rates were increased at a rate of 5% across blocks. When accuracy fell below 80%, the presentation rate was decreased, so that task adaptivity followed a similar step function as in the WM training. Participants were encouraged to practice speeded decisions in each of the linguistic tasks while maintaining high accuracy. A “speed level” score, derived from change in presentation rate from the initial training block, was provided after each block, so that participants could monitor their progress from the first block of the first session to the end of the training, as in the WM training protocol.

### Assessment Battery

The cognitive battery, administered at pre-test and post-test, was chosen to target both complex WM performance as well as measures of off-line language performance. Language outcome measures included assessments of sentence processing and discourse memory, which were themselves graded in terms of their reliance on WM (Stine and Wingfield, 1990; Jefferies et al., 2004; Stine-Morrow et al., 2008), as well as a measure of verbal fluency. At post-test only, a survey to gauge group differences in expectations for cognitive change was administered based on a survey designed by Boot et al. (2013).

#### Complex Verbal Working Memory

Four complex WM tasks were administered using the Psychophysics Toolbox in MATLAB (Brainard, 1997), adapted from the CogToolbox (Fraundorf et al., 2014). In all four tasks, participants made a series of judgments about each item in a set of verbal stimuli and then, after the set, recalled information related to each item within that set. Alternate forms of each task were administered at pre-test and post-test. In the (1) *reading span* and (2) *listening span* tasks (Daneman and Carpenter, 1980; Stine and Hindman, 1994), participants read or listened to a set of simple declarative sentences (e.g., “A book is often found in a library”), and judged whether the sentence was true or false. Additionally, participants were asked to recall the sentence-final words (e.g., library) after each set. The number of sentences per set increased with progress through the task (until eight sentences per set or when the participant could no longer recall each of the target words in a set successfully). If the participant could not recall all items at a particular set size, a second trial was administered. If the participant could not recall all items within the second trial within that set, the test would terminate (cf. Stine-Morrow et al., 2001; Waters and Caplan, 2003; Payne et al., 2014a). The score was the number of target words recalled from the highest set with no errors, plus a fraction reflecting the proportion of correctly recalled words on the set with an error. The listening span used the same administration and scoring, except that the sentences were presented in the auditory modality. For the reading span task, the minimum sentence presentation was 1s and the maximum was 7s. In both the sentence and reading span tasks, the maximum time to make true/false judgments was 2 s. In the (3) *operation span* task (Turner and Engle, 1989; Conway et al., 2005), the participant was cued with a series of three-term math problems (e.g., is [8/2] – 1 = 3; True), followed by a letter (e.g., c) to hold in memory after each problem. Following each problem-item set, the participant recalled the set of letters in the order in which they were presented. Fifteen sets were presented randomly, with set size ranging between 3 and 7. Because there are individual differences in the amount of time participants take to solve arithmetic problems, the presentation rate during the memory test was set on a subject-by-subject basis by using a baseline calibration period (see Unsworth et al., 2005). Prior to the onset of the memory task, participants completed a practice block solving math problems without the memory task. The average time it took to solve each problem was calculated separately for each subject, and the maximum presentation rate was calculated as the subjects mean calibration time plus 2.5 *SD.* The total score was the total proportion of correct items in the correct position across all sets (Unsworth et al., 2005). In the (4) *Minus-2 span task* (Waters and Caplan, 2003), participants were presented with a string of digits one at a time with an SOA of 1 s and cued to produce the series with two subtracted from each digit (e.g., [8, 4, 3, 9] to [6, 2, 1, 7]). The total score was the total proportion of correct items in the correct positions across all trials (Waters and Caplan, 2003). Each span task was preceded by practice at the lowest set size. Note that the selected WM span measures vary with respect to their overlap with surface features (e.g., secondary judgment task, source of elements to recall, scoring criteria, set size order, cf. Was et al., 2011).

#### Sentence Memory

An immediate recall task (Zelinski and Lewis, 2003; Stine-Morrow et al., 2008) was administered in which participants read eight 18-word sentences with presentation time self-paced, and immediately recalled each sentence for later transcription and scoring. Recall was scored as the proportion of individual words correctly recalled (e.g., Potter and Lombardi, 1998; Gilchrist et al., 2008), which for these brief single sentences, was found to correlate very strongly with propositional recall scoring (*r* = 0.91) (Kintsch and van Dijk, 1978; Stine-Morrow et al., 2008). Alternate sentence sets of equivalent difficulty were presented at pre-test and post-test.

#### Syntactic Comprehension

Participants read a series of sentences and answered a simple a yes/no comprehension question after each sentence. Comprehension was assessed for three different types of syntactic complexity that are known to cause comprehension difficulty among older adults, and have been suggested to increase load on WM capacity: (1) *garden-path* syntactic ambiguities, (2) long-distance relative-clause dependencies (Bartek et al., 2011), and (3) object-relative clauses (see **Table 2** for examples)^1^. Sentences were counterbalanced across conditions at each testing occasion, so that each sentence was equally represented in the high- and low-demand conditions. At pre-test and post-test, participants read 20 items from each sentence set (10 low complexity, 10 high complexity), resulting in a total of 60 sentence-question pairs at each measurement occasion.

**Table 2:** Example items in sentence comprehension test as a function of syntactic demand.

Sentence set	Complexity	Sentence
GP	Low	While the man hunted, the deer that was brown and graceful ran into the woods.
GP	High	While the man hunted the deer that was brown and graceful ran into the woods.
SR/OR	Low	The farmer that knew the barber asked for a loan.
SR/OR	High	The farmer that the barber knew asked for a loan.
LDD	Low	The administrator who the nurse supervised scolded the medic for being late.
LDD	High	The administrator who the nurse who was from the clinic supervised scolded the medic for being late.


*GP, garden path; SR/OR, subject/object relative clause; LDD, long distance dependency.*

#### Discourse Comprehension and Memory

The Nelson-Denny Standardized Reading Comprehension subtest, to assess general reading comprehension ability, consists of eight prose passages and 36 multiple-choice questions. Participants were given 20 min to read the passages and answer the questions. Alternate forms were administered at pre-test and post-test. In the Rivermead Behavioral Memory Task Paragraph recall subtest (Wilson et al., 2003), participants listened to a short narrative for immediate recall. Production was coded and scored for the number propositions correctly recalled.

#### Verbal Fluency

Verbal fluency was assessed with the FAS phonemic fluency task (Benton and Hamsher, 1978). In this task, participants were given a letter (at pre-test “F”, “A”, and “S”) and asked to produce as many words that they could think of that begin with that letter for 60 s. A total score is calculated as the sum of unique words correctly produced across the three trials. This task has been shown to be highly predictive of general cognitive status (Kemper and McDowd, 2008) as well as predictive language comprehension (Federmeier, 2007) in older adults. An alternate form, the BDT, was used at post-test (Strauss et al., 2006).

#### Perceptions of Training Benefits

A 14-item survey to assess individuals’ expectations for the effects of training (cf. Boot et al., 2013) was administered at the end of the post-test session. Items probed whether (1) they perceived general improvement in cognition as a function of training (e.g., “I believe that iTrain helped improve my cognition”), and (2) they improved on specific tasks (e.g., for the Listening Span task, “You completed a task called Listening Memory. In this task, you heard a series of sentences and you were asked to judge if the sentences were true or not. You were also asked to remember the last word of each of the sentences in that section in order. Do you believe that iTrain helped lead to better performance on this task?”). Participant’s read each statement and then were asked to endorse those statements on a Likert scale from 1 (Strongly Disagree) to 5 (Strongly Agree).

### Procedure

At the onset of the study, all participants completed the cognitive battery in a single 3-h laboratory session. Following the pre-test battery, participants were given an iPad 2 tablet computer containing either the complex WM training software (treatment group) or the active control training software, based on random assignment. Testers instructed participants on procedures for completing each of the tasks in the training program, and participants were given the opportunity to practice the tasks in the lab until they understood each task completely. Participants returned to the lab at the end of the training for post-test. The testing was single blind, as testers were aware of the random assignment condition. However, testing sessions were designed to minimize the amount of contact with the participant, and testers were instructed to provide no identifying information regarding the training program or the study hypotheses.

## Results

A series of linear mixed effects models were used to test for the effects of the intervention on each outcome measure. Analyses focused on effect size estimation and quantification of the precision of these effects via confidence intervals (Kelley and Preacher, 2012; Lakens, 2013; Cumming, 2014). Effect sizes and 95% profile confidence intervals of the critical Training Group (Control vs. Training) × Time (Pre-test vs. Post-test) interactions were estimated via restricted maximum likelihood estimation, with random intercepts specified for subjects, and by-subject random slopes for the within-subject Time factor. For the syntactic comprehension data, the critical interaction was a Group × Time × Sentence Type effect and, for these models, the Time × Sentence Type interaction was additionally modeled as a random slope (see Barr et al., 2013; Bates, under review for discussions on the treatment of random slopes). Note that, following an intention-to-treat protocol, all participants were invited back for post-testing and included in all analyses, regardless of the number of sessions that were completed. Thus, models were fit to all available data for each outcome. Treatment coding was used for all fixed-effects factors and statistical inference was limited to the critical interactions that would provide statistical support for group differences in the change in each outcome from pre-test to post-test. Because sample sizes are small, supplemental non-parametric analyses were conducted using a robust bootstrapping approach as described by Kirby and Gerlanc (2013), to estimate the standardized effect size and precision of group differences in change in the outcome measures.

### Perceptions of Training Benefit

**Table 3** presents mean rating endorsements for both general improvement in cognitive ability, as well as improvement across specific tasks. Overall, average endorsement rates ranged from neutral to positive. Importantly, there was no difference between the treatment and control groups in expectations that they improved in overall cognition following training (*b* = 0.13; 95% CI [-0.15, 0.41]). There was a trend for the WM tasks to show self-reports of greater improvement in the treatment group relative to the control. However, only for one task—the minus-2 span task— did the group difference reach statistical significance, though this effect was quite small (*b* = 0.65; 95% CI [0.004, 1.29]). Importantly, the language tasks showed no evidence of differential expectation for improvement between the control and training groups, with the trend going in the direction of greater perceived improvement in language tasks in the control group (see **Table 3**). Thus, it is unlikely that the effects of training on language outcomes reported below could be attributed to differential expectations for improvement in the training group relative to the active control group.

**Table 3:** Results from post-test expectation survey.

	Control		Training		Group difference	
			
	*M*	*SE*	*M*	*SE*	*Diff*	*95% CI*
Reading span	3.23	0.21	3.55	0.21	0.31	[-0.22, 0.84]
Listening span	3.15	0.18	3.5	0.18	0.35	[-0.11, 0.81]
Minus-2 span	3.23	0.21	3.89	0.21	0.65	[0.004, 1.29]
Operation span	2.91	0.16	3.47	0.16	0.56	[-0.03, 1.15]
Syntactic comprehension	3.38	0.16	3.23	0.16	-0.16	[-0.69, 0.27]
Text memory	3.23	0.23	3.00	0.23	-0.23	[-0.23, 0.36]
Reading comprehension	3.62	0.29	3.27	0.29	-0.34	[-1.06, 0.38]
Overall cognition	3.34	0.12	3.47	0.10	0.13	[-0.15, 0.41]


*Items on a Likert scale with 1 indicating low endorsement of training benefit and 5 indicating high endorsement of training benefit. Both groups rated expectations of improvement slightly above neutral (3) across nearly all survey items. 95% confidence intervals containing 0 indicate differences than are not statistically significant at *p* < 0.05. The only item that reached a significant difference was for the Minus-2 span task.*

### Training-Related Changes in WM Performance

**FIGURE 2:**
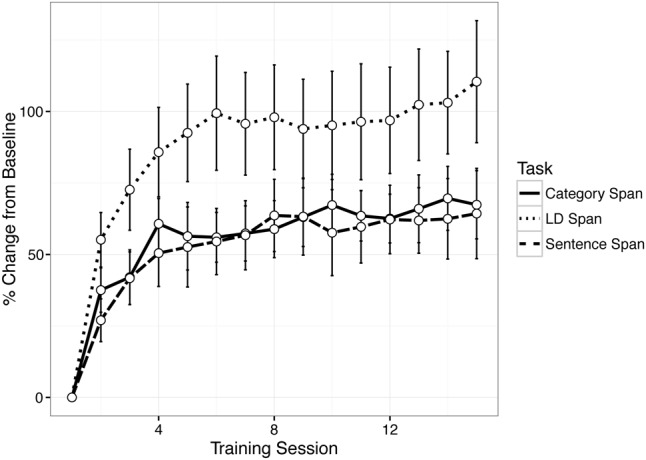
Training group percent change from baseline in working memory (WM) span on three training tasks over 15 sessions.

Trial-level performance was collected by the iPad over the course of training, enabling us to compute the average span score for individuals at each session for each span task. Based on participants who completed at least 80% of the training (*n* = 21), **Figure 2** plots the session-to-session effects of WM training on performance gains for each of the three verbal WM tasks. Raw scores on the span tasks were converted to a metric of percent change from baseline assessment (i.e., their average score on the first day of training) in order to assess the relative degree of improvement from baseline on a similar scale for each span task (e.g., Chein and Morrison, 2010). On average, training gains followed a non-linear trajectory, with larger improvements in early sessions. Indeed, the largest improvements across the three tasks occurred from session 1 to session 2. Over the 15 sessions, trainees showed an approximate 60% peak training improvement from baseline on the category and sentence span tasks and more than doubled their span performance on the lexical decision span task relative to their performance on the first day of training.

**FIGURE 3:**
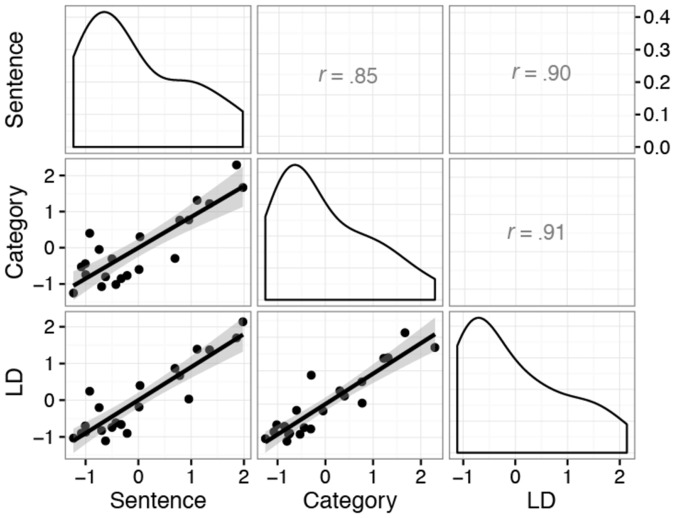
Scatterplot matrix of the relationship between training improvements over 15 training sessions across each of the three WM training tasks. The lower diagonal cells are bivariate scatterplots and best-fit linear functions of the relationship between WM training gains across each pair of tasks. The upper diagonal presents the corresponding Pearson correlation coefficient for each pair of training tasks. The diagonal cells plots the estimated probability density functions of the individual training improvements in each task. Note that training improvements were *z*-score standardized, so that 0 represents average training improvement, and a 1 unit increase represents an approximate 1 *SD* improvement.

To examine individual differences in performance, we calculated subject-specific learning curves, expressed as the percent of change from baseline performance. To accommodate the non-linearity in training gains, a natural cubic smoothing spline was fit to the training data for each participant. Following this, the area under the cubic spline curve was estimated over the training period separately for each participant (using an adaptive quadrature algorithm via the MESS package in R; Venables and Ripley, 2002) as a summary index of non-linear training gains in each task for each individual across the 15 sessions. **Figure 3** plots the bivariate scatterplot matrix among training gains for the three span tasks. With correlations above 0.85, it is apparent that training-related improvements on the trained memory span tasks clustered together tightly, suggesting that training-related improvements occurred broadly and to a similar degree across all tasks, and were thus not likely due to isolated to task-specific strategy development (which would attenuate the correlation among the training-related improvements).

### Transfer to Working Memory and Language

**FIGURE 4:**
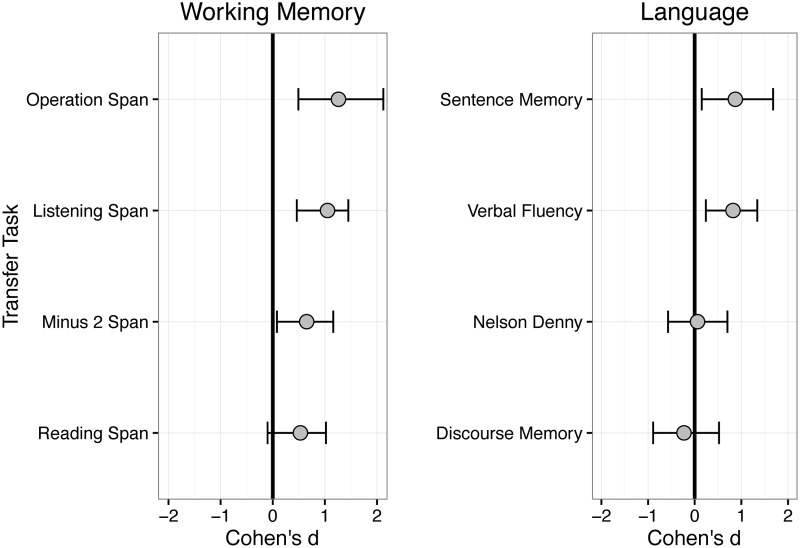
Effect sizes (Cohen’s *d*) and 95% bootstrapped confidence intervals of the training group × time interaction for WM **(Left)** and language **(Right)** measures from the neuropsychological test battery. More positive values indicate a greater change from pre-test to post-test for the training group relative to the control group.

**Table 4** presents pre-test and post-test mean scores and difference scores for the WM and language tasks administered in the cognitive test battery separately for the control group and treatment groups. Note that, following an intention-to-treat approach, these results reflect all available data at pre-test and post-test, regardless of participants’ training adherence. **Figure 4** presents summary bootstrapped effect sizes of the group differences in change in each of the tasks (e.g., the Group × Time interaction) in units of standard deviation change (Cohen’s *d*) separately for the WM and language tasks. Larger values indicate a positive change from pre-test to post-test that was larger for the treatment group than the control group. Appendix A presents correlations between the WM measures and language tasks at baseline.

**Table 4:** Pre-test, post-test, and change in working memory and language measures in control and treatment groups.

	Control						Treatment					
		
	Pre-test		Post-test		Δ		Pre-test		Post-test		Δ	
						
	*M*	*SE*	*M*	*SE*	*M*	95% CI	*M*	*SE*	*M*	*SE*	*M*	95% CI
**Verbal working memory**												
(1) Reading span	2.74	0.25	2.98	0.34	0.22	[-0.20, 0.65]	2.85	0.20	3.70	0.22	0.84	[0.28, 1.40]
(2) Listening span	3.50	0.37	2.88	0.31	-0.64	[-1.31, 0.03]	3.68	0.16	4.40	0.19	0.72	[0.24, 1.18]
(3) Operation span	0.40	0.07	0.44	0.06	0.02	[-0.04, 0.09]	0.39	0.05	0.63	0.05	0.30	[0.19, 0.41]
(4) Minus-2 span	0.58	0.05	0.63	0.06	0.01	[-0.03, 0.05]	0.63	0.04	0.76	0.02	0.12	[0.03, 0.21]
**Language transfer**												
(5) Nelson-Denny	0.87	0.02	0.82	0.04	-0.06	[-0.13, 0.01]	0.86	0.02	0.81	0.05	-0.05	[-0.11, 0.02]
(6) Verbal fluency	41.00	2.47	42.71	2.07	0.71	[-3.15, 4.56]	43.82	2.22	51.36	3.00	7.55	[3.96, 11.13]
(7) Sentence memory	0.66	0.04	0.71	0.03	0.04	[-0.01, 0.07]	0.65	0.02	0.77	0.02	0.12	[0.08, 0.16]
(8) Discourse memory	0.46	0.04	0.44	0.04	-0.02	[-0.86, 0.43]	0.52	0.03	0.47	0.03	-0.05	[-0.11, 0.01]


*Reading span and listening span scores are the number of target words recalled from the highest set with no errors, plus a fraction reflecting the proportion of correctly recalled words on the set with an error. Operation span, Minus-2 span, Nelson-Denny, sentence memory, and discourse memory are scored as proportion correct. Verbal fluency is total number of words produced. Δ is the difference between pre-test and post-test. 95% Confidence Intervals containing 0 indicate differences than are not statistically significant at *p* < 0.05. There were no significant differences between Control and Treatment groups on any measure at baseline.*

#### Working Memory

There were no reliable baseline differences in any WM task between treatment and control groups (all *t*’s < 1, see **Table 4**). As can be seen in **Table 4** and **Figure 4**, there was evidence for training-related improvements in verbal WM. All four WM tasks showed effect sizes larger than *d* = 0.50, indicating at least an approximate half standard deviation difference between the treatment and control groups in change in WM. The average effect size of training collapsing across the four tasks was *d* = 0.87. However, 95% confidence intervals were quite large for all of the tasks, suggesting substantial individual differences in responsiveness to the intervention (cf. Payne et al., 2012b). Results from linear mixed-effects models fit to each task showed reliable Group × Time interactions for Listening Span (*b* = 1.32, 95% CI [0.54, 2.12]), Operation Span (*b* = 0.24, 95% CI [0.08, 0.40]), and Minus-2 Span (*b* = 0.10, 95% CI [0.02, 0.18]), indicating greater improvement in span for the training group relative to the control group. Of the four tasks, Reading Span showed the weakest effects, with a negative lower-bound on the bootstrapped 95% confidence interval. Consistent with the bootstrapped effect sizes, the linear mixed-effects model revealed a non-significant Group × Time interaction (*b* = 0.61, 95% CI [-0.12, 1.34]) for reading span only.

#### Language Outcomes

There were no reliable baseline differences between treatment and control groups on any measure (all *t*’s < 1.3, see **Table 4**). As shown in **Table 4** and **Figure 4**, verbal fluency and sentence memory showed evidence of improvement from WM training relative to the control group. The Group × Time interaction was significant for verbal fluency (*b* = 6.57, 95% CI [1.30, 11.79]) and sentence memory (*b* = 0.08, 95% CI [0.02, 0.14]), such that the WM training group showed a larger increase compared to the control group. In contrast, the two tasks assessing discourse understanding and memory, the Rivermead and the Nelson-Denny tasks, showed no evidence of training-related improvements as the Group × Time interaction was not reliable for either the Nelson-Denny (*b* = 0.002, 95% CI [-0.11, 0.12]) or the Rivermead discourse memory task (*b* = -2.85, 95% CI [-11.61, 5.91]).

**FIGURE 5:**
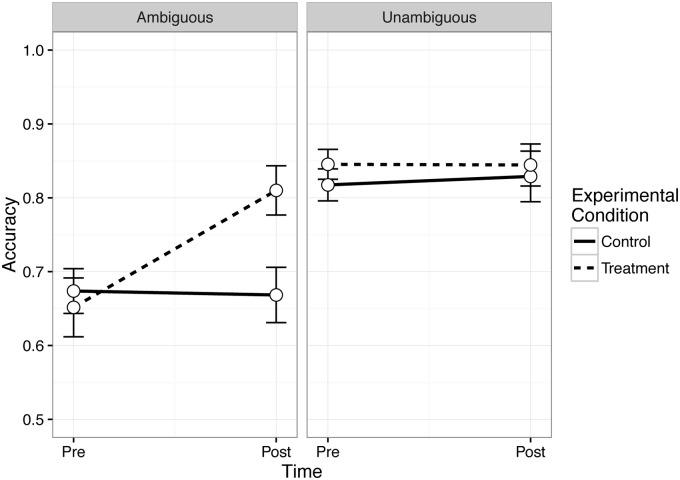
Effects of training on syntactic ambiguity resolution accuracy.

Finally, WM training showed isolated effects in improving comprehension of ambiguous syntactic forms. **Table 5** presents pre-test and post-test mean scores for the low- and high-demand conditions of each of the three syntactic comprehension sets separately for the control group and treatment groups. For the garden-path sentences, a reliable ambiguity effect was observed at baseline (*b* = 0.14, 95% CI [0.06, 0.23]), such that ambiguous sentences had poorer accuracy than unambiguous sentences. In the model testing training effects on ambiguity resolution, a reliable Syntactic Demand × Time × Treatment interaction was found (*b* = -0.18; 95% CI [-0.34, -0.01]), indicating that there were training group differences in the change in accuracy from pre-test to post-test that differed for the ambiguous sentences compared to the unambiguous sentences. This interaction is depicted in **Figure 5**. As can be seen, accuracy was high across both training groups for syntactically unambiguous sentences at pre-test and post-test. For the ambiguous sentences, however, sentence comprehension is poorer at baseline and only the WM training group showed improvement in accuracy from pre-test to post-test.

**Table 5:** Syntactic comprehension in control and treatment groups at pre-test and post-test.

	Control				Treatment			
		
	Pre-test		Post-test		Pre-test		Post-test	
				
	*M*	*SE*	*M*	*SE*	*M*	*SE*	*M*	*SE*
**Garden path**								
Unambiguous	0.82	0.02	0.83	0.03	0.85	0.02	0.84	0.02
Ambiguous	0.67	0.03	0.66	0.03	0.65	0.04	0.81	0.03
**Subject-object relative**								
Subject-relative	0.81	0.04	0.79	0.03	0.87	0.03	0.76	0.03
Object-relative	0.68	0.04	0.68	0.04	0.65	0.04	0.74	0.04
**Long-distance dependency**								
Short-distance RC	0.68	0.03	0.71	0.03	0.70	0.04	0.71	0.03
Long-distance RC	0.69	0.05	0.58	0.04	0.73	0.04	0.70	0.02


For the subject and object relative sentences, a reliable OR-cost effect was observed at baseline, such that object-relative sentences had poorer accuracy than subject-relative sentences (*b* = 0.13; 0.04, 0.22). In the model testing training effects on SR/OR comprehension, a small Syntactic Demand × Time × Treatment interaction was observed (*b* = -0.18; 95% CI [-0.35, -0.007]). However, the nature of this interaction was difficult to attribute strictly to training-related improvements in the costs associated with object-relative processing, as the interaction was driven by the WM training group showing a relative improvement in object-relative comprehension from pre-test to post-test (as predicted), but a corresponding decline in improvement in the simpler subject-relative sentences, which is not the expected pattern following WM training.

For the long-distance dependency sentences, surprisingly, there was no reliable LDD-cost observed at baseline (*b* = -0.007; 95% CI [-0.11, 0.10]), as accuracy was approximately equivalent between relative clauses with and without a long-distance dependency introduced between the head noun and the relative-clause verb. Although there was a trend for a Syntactic Demand × Time × Treatment interaction (*b* = -0.21; 95% CI [-0.41, 0.005]) that did not reach statistical significance, like the SR/OR sentences, the nature of the interaction was difficult to attribute to training-related improvements accuracy for the more complex long-distance dependency case (see **Table 5**).

## Discussion

It is often assumed that normative age-related changes in basic cognitive functions such as WM capacity compromise the ability to comprehend language and learn from complex texts. However, nearly all of the evidence for a role of the WM system in language comprehension is derived from correlational studies, which are inherently limited. In order to resolve this causal ambiguity, the current study exploited an experimental design to examine the degree to which cognitive training in verbal WM could transfer to aspects of language comprehension among older adults using a novel home-based cognitive training program.

The data presented in the current study yield important insights into both the nature of the verbal WM system and the degree of plasticity in language comprehension among older adults. Specifically, our results indicated that verbal WM is capable of short-term change in adults over the age of 60 through less than 10 h of home-based training over the course of 3 weeks, and that this training showed some evidence of transfer to untrained verbal memory measures as well as measures of language fluency, language memory, and syntactic comprehension. In summary, our findings suggest that WM is plastic in later adulthood, at least in the short-term. These data, while preliminary in nature, are among the first to indicate that selective aspects of WM-dependent language performance can be modified through targeted practice in WM in older adulthood.

### Home-Based Working Memory Training

The benefits of home-based training via tablet computers include convenience for the participant and a reduction of resources devoted to weekly testing sessions in the lab. Moreover, lab-based training may lead to biased sampling of study participants who are highly mobile, healthy, and able to allocate substantial amounts of time each week to participating in laboratory sessions. In contrast, home-based training is likely to lead to more heterogeneous sampling at both ends of the ability distribution (i.e., high-ability adults with substantial time and scheduling constraints that prevent participating, as well as lower ability and lower-mobility adults), as it reduces the burden on the participant to complete daily lab visits over several sessions.

However, a major component of home-based training is that it requires the trainees to self-administer and self-monitor their training progress throughout the course of the intervention, which may impact training responsiveness. Very few studies have examined the effects of home-based cognitive training in older adults. In two experiments, Wadley et al. (2006) directly compared training gains in a useful-field-of-view training program among healthy older adults in laboratory and home settings. Both groups showed significant improvements in processing speed relative to a control group that underwent no training. However, gains in the home-based group were 74% that of those in a lab-based training condition. These data suggest that self-administration of cognitive training is indeed feasible, though effect sizes may be smaller and more heterogeneous (see Payne et al., 2012b for similar evidence in a home-based reasoning training). Such home-based training is likely to be more sensitive to individual differences in motivational factors, which may directly influence the amount of effort allocated to the training (e.g., Payne et al., 2012b). Note however, that Wadley et al.’s (2006) findings could also be attributed to the adaptive nature of the in-lab training, which was not replicated in the home version. Nevertheless, the data from the current study indicate that self-administration of the WM training is feasible.

A key test of the effectiveness of the training program was the assessment of the degree to which training led to improvements in untrained complex verbal WM span. There was positive evidence for improvement across the complex span tasks measured in the current study, with all four tasks showing at least a half standard deviation improvement in WM for the training group relative to the control, three of the four tasks reaching statistical significance, and a pooled effect size of *d* = 0.87. Thus, the evidence from the current study suggests that home-based training of WM can be effective in improving both trained and untrained complex verbal WM span in the short-term. Note however, that the effects of training on the reading span task did not reach statistical significance despite the fact that this measure appeared to have the highest overlap in surface features to one of the training tasks (the sentence span task) while the same outcome in the auditory modality (listening span) did show reliable evidence for near transfer. One reason for the reduced effect size of training on reading span relative to listening span may be due to the self-paced nature of the reading span measure. Prior studies have found that self-paced administration influences estimated WM capacity, as well as the validity of the measure for predicting higher-order cognition (Friedman and Miyake, 2004; Lépine et al., 2005; Clair-Thompson, 2007; Barrouillet et al., 2008). It may be the case that self-pacing in the processing component of the task influences the magnitude of transfer as well.

### Working Memory and Language Understanding

The primary aim of the current study was to test the degree to which training-related improvements in WM led to improvements in language comprehension in older adults. Adults in the WM training group showed differentially larger improvements in both sentence memory and verbal fluency relative to the active control group. It is perhaps unsurprising that short-term sentence memory showed transfer, as sentence memory performance is highly related to WM (Stine-Morrow et al., 2008; Lewis and Zelinski, 2010; Payne et al., 2012a), and, at least for the reading span task, overlaps to some degree in task demands (MacDonald and Christiansen, 2002; cf. Roberts and Gibson, 2002). However, demonstrating training-related transfer to sentence memory in older adults is critical for at least two reasons. First, although verbal WM and sentence memory share a substantial amount of variance in older adults, this does not necessarily imply that training should result in transfer. Indeed, individual differences in WM and fluid intelligence share upward of 50% of the same variance (Engle, 2010), and yet evidence for transfer of WM training to fluid intelligence has been inconsistent (see Shipstead et al., 2012; Melby-Lervåg and Hulme, 2013, 2016, for reviews). Second, sentence memory shows some of the largest effect sizes for age-related declines among measures of language comprehension and episodic memory (Johnson, 2003; Stine-Morrow et al., 2008). Demonstrating reductions in age-related deficits in language memory is thus quite valuable for future applications in memory remediation in older adulthood.

The demonstration that WM training transferred to verbal fluency indicates that training can lead to transfer to tasks that share very little overlap with the tasks involved in the training. At the same time, interpreting training effects on verbal fluency are complicated by the fact that tasks such as the *FAS* are used in both research and clinical settings to index a range of theoretically different cognitive functions including executive functioning (Mayr and Kliegl, 2000), semantic processing efficiency (Troyer et al., 1997), frontal-lobe mediated generative language production (Federmeier, 2007), and lexical knowledge (Nagels et al., 2012). Future work should focus on the role that WM training plays in improving executive control components related to aspects of language production and semantic processing. Nevertheless, given the strong relationship between fluency and language comprehension and production in older adulthood (Federmeier et al., 2002, 2010; Federmeier, 2007; Wlotko et al., 2012), such findings are promising from both applied and basic perspectives.

Two tasks tapping discourse comprehension showed no evidence of transfer of training gains: the Nelson-Denny reading comprehension task and the Rivermead behavioral memory task, a measure of discourse memory (see Payne et al., 2014b). While this may be surprising given that prior work has shown that reading comprehension and discourse recall are correlated with WM, one explanation is that age-related declines in discourse understanding are actually quite rare (Stine and Wingfield, 1990; Radvansky, 1999; Radvansky and Dijkstra, 2007). To some extent, this may be due to the reliance of discourse comprehension on the establishment of a situation model, a level of understanding that is robust to cognitive aging (Radvansky and Dijkstra, 2007; Stine-Morrow and Radvansky, in press). Under this account, older adults can rely on situational representations as a compensatory mechanism in order to maintain comprehension despite reduced memory resources. However, for the context-independent sentence memory task, where it is less likely that a situational representation can be established, WM effects are larger, and effects of training are found.

Finally, we tested the degree to which syntactic comprehension accuracy was modulated by WM training. Results were mixed. Sentences that were unambiguous but more syntactically complex (e.g., SR/OR and LDD sentence sets) did not produce the expected pattern of training-related improvements. Only in syntactically ambiguous garden path sentences was there positive evidence for WM-specific-improvements in comprehension of more syntactically difficult sentences. Both the treatment and control groups showed the canonical garden-path ambiguity effect in comprehension (Christianson et al., 2001, 2006) at baseline. At post-test, only the WM training group showed evidence for reduced ambiguity effects on comprehension. This effect was driven by a selective increase in comprehension for the more demanding syntactically ambiguous items. Note that these findings are similar to those of Novick et al. (2013) and Hussey et al. (2016) who have found evidence that younger adults trained on the *n*-back task with lures showed improvements in comprehension of similar garden-path ambiguities.

One explanation is that WM affords the capacity to maintain multiple alternative syntactic representations of ambiguous phrases, which can be directly accessed at the point of disambiguation. Low-span readers are unable to maintain multiple syntactic representations, and therefore commit to one interpretation, causing subsequent difficulties when they must revise their incorrect interpretation (MacDonald et al., 1992; Kemper et al., 2004). Consistent with this account, Christianson et al. (2006) showed evidence for a robust negative correlation between verbal WM span and the probability of incorrectly interpreting garden path sentences in older adults. These findings suggest that older adults with low WM have particular difficulties in revising an initially incorrect interpretation (see also Payne et al., 2014a for similar evidence in syntactic attachment ambiguities). The training data presented here corroborate prior correlational results and extend these by suggesting that the WM system subserving ambiguity resolution is plastic and is responsive to memory training.

### Limitations and Future Research

The primary limitation in the current study is that the small sample size limited our power to detect small-to-moderate effect sizes. The issue of small sample sizes is widespread in the WM training literature. This issue is largely driven by the severe resource constraints associated with conducting adequately powered cognitive training studies in special populations due to issues with staffing, recruitment, retention, maintenance of intention-to-treat protocols, and additional costs of conducting longitudinal randomized controlled trials with large sample sizes. One way in which sample sizes may be increased without substantially increasing costs and resources is through home-based training and assessments, as these approaches require fewer resources to be allocated to each individual subject for daily laboratory visits. Thus, one goal of this work is to illustrate that home-based training is a feasible and valid option for future studies and may be able to help move toward scaling up studies to optimally powered sample sizes to detect more nuanced and reproducible effects of training. Because the current study is our first attempt at targeting language comprehension in older adults using home-based WM training, the results should be interpreted with caution. Future planned work will aim to replicate these results with larger and more diverse samples and continue to follow best practices for cognitive intervention, including pre-registration and examining follow-up and maintenance effects (cf. Simons et al., 2016).

Despite our limited sample size, several advances were made in the current study to meet the criteria of a randomized controlled trial, as laid out in the CONSORT statement. Great care was taken to evaluate the effects of iTrain against an appropriate control group in the context of a literature in which inadequate control groups negatively impact many studies. Because treatment and no-contact control groups are not matched on their expectancies to improve, differential change can be attributed to Hawthorne effects, in which task-related expectancy to improve drives motivational factors to improve performance at post-test. Even in studies with so-called “active” control groups, different groups may vary substantially in their expectations for improvement generally as well as on specific tasks (Boot et al., 2013). In this study, we adopted a “component control” design to keep control and treatment groups as well matched as possible. Indeed, post testing surveys revealed that individuals in both groups had similar endorsement of perceived training improvements. That only moderate perceived change was found in the presence of observable improvement suggests that these effects are not likely attributable to so-called “Hawthorne” effects. In addition, an intention-to-treat approach was used, in order to downwardly bias effect sizes with differential drop from the training (Hollis and Campbell, 1999). However, because the home-based training resulted in such high retention, the issue of differential drop-out causing the observed training benefits is not plausible.

Despite the relative breadth of the measurement battery for assessing language, it was designed to primarily tap into comprehension processes and not language production. However, there is a growing literature posing a strong relationship between WM, WM limitations (e.g., through aging and brain damage), and language production mechanisms (Acheson and MacDonald, 2009; Martin and Slevc, 2014). Indeed, two tasks in the neuropsychological battery—the verbal fluency and the sentence memory tasks—involved verbal production and also showed the strongest evidence of training benefits, despite production *per se* not being the critical theoretical component of these measures. Thus, future work may benefit from more thoroughly targeting language production outcomes.

## Conclusion

The contributions of this study are two-fold. First, based on a research design that minimized the role of expectancy effects (Boot et al., 2013), our results suggest that verbal WM among older adults is responsive to home-based training, at least in the short-term. With fewer than 10 h of home-based practice with tasks exercising the simultaneous management of verbal operations and storage over the course of 3 weeks, training effects transferred to untrained verbal WM measures. Second and most importantly, WM training lead to selective improvements in measures of language fluency, sentence memory, and syntactic ambiguity resolution, implying that WM may be a critical resource for these aspects of language performance in older adulthood. These findings are among the first to indicate that selective aspects of language performance can be modified through targeted home-based practice in WM in older adulthood. This is not only of theoretical import in defining the cognitive architecture of language processing across the lifespan (e.g., Just and Carpenter, 1992; Stine-Morrow and Payne, 2016), but also suggests applications for improving cognitive functioning among older adults in significant ways (Stine-Morrow and Basak, 2011). Because age-related declines in language comprehension and memory can have far-reaching effects as adults navigate the ordinary demands of work, family, and health (e.g., Morrow et al., 2006; Chin et al., 2015), the development of pathways to mitigate such deficits offers promise for promoting late-life well-being.

This study is motivated by a specific model of vertical transfer in which a skill (language comprehension) was improved by exercise of a component theorized to be a core process constraining this skill (verbal WM). As described above, the cognitive training literature has generally shown relatively narrow transfer of cognitive training across cognitive domains in near transfer tasks. Yet, it is common for training programs to adopt a very broad cognitive battery and predict broad-based changes in cognition, under the rationale of “use it or lose it,” without a specific model of what is exactly is being used or what capability will not be lost by using it. Given what is known from existing literature (cf. Simons et al., 2016), it is plausible that training programs will lead to very specific improvements across cognitive domains in transfer tasks that are subserved by the core mechanisms being exercised in the training tasks. A goal of future work must be to develop sound theories of the cognitive architecture of meaningful activities, as well as credible training and transfer tasks to operationalize those theories. Only in this way can cognitive training be used as a method to target specific mechanisms and test mechanistic accounts of theoretical models (Baltes and Kliegl, 1992; Hussey and Novick, 2012; Lindenberger et al., 2017). Thus, although transfer of cognitive training is a controversial area (see open-letter statements by A Consensus on the Brain Training Industry from the Scientific Community, 2014 and cognitivetrainingdata.org), our view is that the current study contributes to a literature aimed at using training-related cognitive plasticity as a tool for examining basic questions about cognitive architectures and functions rather than as a tool to reverse or slow generalized cognitive decline or substantially alter intellectual functioning (Simons et al., 2016; National Academies of Sciences, Engineering, and Medicine, 2017).

## Ethics Statement

University of Illinois Institutional Review Board. Participants had completely voluntary participation and chose to sign up for the study of their own volition. All appropriate procedures were followed to ensure subject confidentiality and privacy, including voluntary participation, freedom to withdraw, and informed consent. Prior to participation in the study, informed consent was obtained from participants, which involved verbally explaining the research, risks and benefits, and confidentiality. Risk level for this study was deemed to be no more than minimal risk, meaning that the probability and magnitude of harm or discomfort were not greater than those ordinarily encountered in daily life.

## Author Contributions

BP and ES-M designed the research. BP performed the research and analyzed the data. BP and ES-M wrote the paper.

## Conflict of Interest Statement

The authors declare that the research was conducted in the absence of any commercial or financial relationships that could be construed as a potential conflict of interest.

## Footnotes

1 Participants read these sentences while having their eye-movements monitored to examine changes in on-line language processing. The eye-tracking data showed no reliable changes over time nor effects of the intervention.
