# Supplementary material for: The Effects of Home-Based Cognitive Training on Verbal Working Memory and Language Comprehension in Older Adulthood
Source: Front Aging Neurosci. 2017 Aug 8;9:256. doi: 10.3389/fnagi.2017.00256 (PMC5550674; doi:10.3389/fnagi.2017.00256)

Appendix A. Graphical Depiction of Working Memory Measures and Language Outcomes at Baseline. Below diagonal are scatterplots with best-fit linear trend lines. Above diagonal are Pearson correlations. Diagonal is probability density of measure. Correlations exceeding .31 (uncorrected) are statistically significant at *p* < .05.


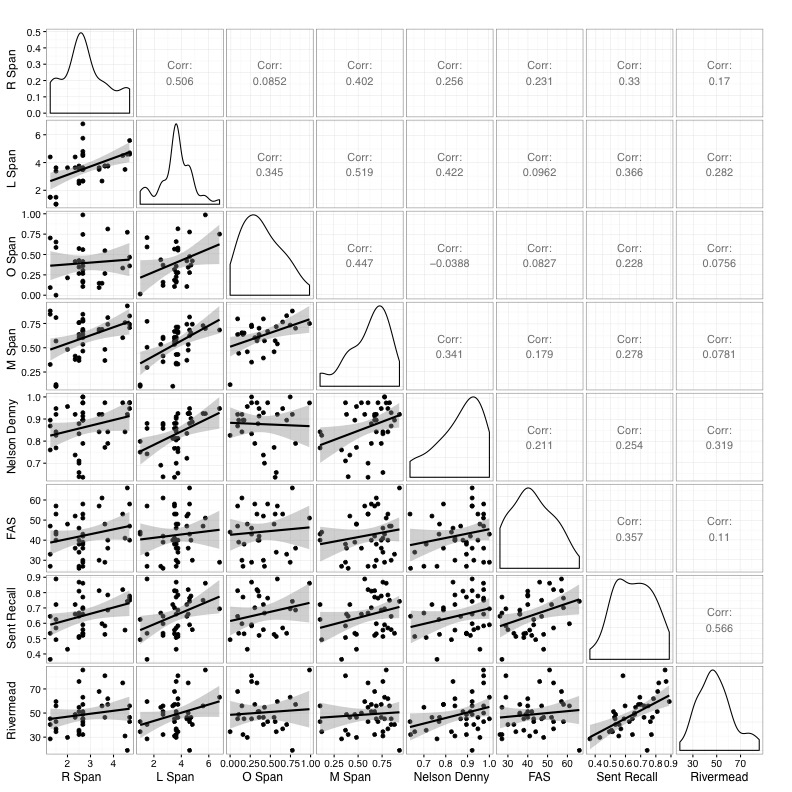

Supplement: Supplementary file 1 [file Data_Sheet_1.docx]
